# Buffalo Medical and Surgical Association

**Published:** 1882-12

**Authors:** Clayton M. Daniels

**Affiliations:** Secretary


### Society Reports.

### BUFFALO MEDICAL AND SURGICAL ASSOCIATION.

*Regular Meeting, November 6th, 1882.*

Vice-President, Dr. J. Cronyn, in the Chair. Dr. C. M. Daniels, Secretary.

Present—Drs. Cary, Stockton, Wyckoff, Hartwig, Hauenstein, Barrett, Harvey, Fowler, Bartlett, Strong, and, by invitation, Drs, Hill, Frank, Wall, Clark, Frederick, Long, Pryor and Thornton.

Upon motion, the reading of the minutes of the last regular meeting was dispensed with.

Application for membership was received from Dr. Chas. A. Wall, who was duly elected.

The Secretary read a letter from the essayist for the evening, Dr. W. S. Tremaine, stating that it would be impossible for him to attend, on account of illness. Upon motion, it was decided to await his recovery before discussing the subject, "Strictures of the Urethra," that he was to present.

*Prevailing Diseases*—Dr. Hauenstein had nothing special to report, except occasional cases of scarlatina and diphtheria.

Dr. Bartlett presented a similar report, and had noted several cases having enlarged cervical glands, also that dropsical tendencies were more than usually prevalent, even under the best of hygienic surroundings.

*Miscellaneous Business*—Dr. Hartwig asked that where patients were presented before the Association, such might be seen before the regular order of business.

Dr. Cronyn replied that he considered the best way would be for those desiring to present patients to advise the Secretary in advance, and special action could be taken at any individual meeting which would not necessitate any change in the by-laws.

Dr. Wyckoff asked why the hour of meeting had been changed to a quarter past eight, instead of earlier as had been the custom heretofore.

The Secretary stated that it was deemed advisable to make the change to accommodate those physicians who had the usual evening office hours, from seven to eight o'clock.

Clayton M. Daniels, *Secretary.*

